# Methylene Blue Dye Photocatalytic Degradation over Synthesised Fe_3_O_4_/AC/TiO_2_ Nano-Catalyst: Degradation and Reusability Studies

**DOI:** 10.3390/nano10122360

**Published:** 2020-11-27

**Authors:** Seyedehmaryam Moosavi, Rita Yi Man Li, Chin Wei Lai, Yusliza Yusof, Sinyee Gan, Omid Akbarzadeh, Zaira Zaman Chowhury, Xiao-Guang Yue, Mohd RafieBin Johan

**Affiliations:** 1 Nanotechnology & Catalysis Research Centre (NANOCAT), Institute for Advanced Studies (IAS), University for Malaya (UM), Level 3, Block A, Kuala Lumpur 50603, Malaysia; cwlai@um.edu.my (C.W.L.); lyzaa@um.edu.my (Y.Y.); omid@um.edu.my (O.A.); dr.zaira.chowdhury@um.edu.my (Z.Z.C.); mrafiej@um.edu.my (M.R.J.); 2 Sustainable Real Estate Research Center, Hong Kong Shue Yan University, North Point, Hong Kong 999077; ymli@hksyu.edu; 3 Publication Unit, Information Technology and Corporate Services Division, Malaysian Palm Oil Board (MPOB), Kajang 43000, Selangor, Malaysia; gan@mpob.gov.my; 4 School of Sciences, European University Cyprus, 1516 Nicosia, Cyprus; x.yue@external.euc.ac.cy; 5 Faculty of Engineering and Technology, Siksha ‘O’ Anusandhan University, Bhubaneswar 751030, India

**Keywords:** photo-catalytic degradation, activated carbon, dye, reusability, magnetic separation

## Abstract

In this study, activated carbon (AC) from coconut shell, as a widely available agricultural waste, was synthesised in a simple one-step procedure and used to produce a magnetic Fe_3_O_4_/AC/TiO_2_ nano-catalyst for the degradation of methylene blue (MB) dye under UV light. Scanning electron microscopy revealed that TiO_2_ nanoparticles, with an average particle size of 45 to 62 nm, covered the surface of the AC porous structure without a reunion of its structure, which according to the TGA results enhanced the stability of the photocatalyst at high temperatures. The photocatalytic activities of synthesised AC, commercial TiO_2_, Fe_3_O_4_/AC, and Fe_3_O_4_/AC/TiO_2_ were compared, with Fe_3_O_4_/AC/TiO_2_ (1:2) exhibiting the highest catalytic activity (98%). Furthermore, evaluation of the recovery and reusability of the photocatalysts after treatment revealed that seven treatment cycles were possible without a significant reduction in the removal efficiency.

## 1. Introduction

Numerous techniques for removing a synthetic dye from aqueous effluents to reduce their effects on the environment have been designed and developed, including adsorption, Fenton-like reactions, activated sludge, membranes, reductive degradations using zero-valent iron, and photo-catalysis. Among these methods, methods based on membranes and adsorption have shown high removal efficiencies (>90%) [1] but have several disadvantages, as these methods only can trap and retain the impurities, not destroy them. Moreover, the used adsorbents and rejected water from membrane processes are categorised as secondary contaminants [2].

Photocatalysis, in which the clean, safe, and inexhaustibly abundant energy of the sun can be harnessed, is a major advance in this field. Dyes such as synthetic organic compounds and common pollutants in wastewaters are widely used in many industries, such as textiles, cosmetics, food, printing, plastics, and leather. Disposal of dye pollutants into the water sources inhibits sunlight permeation into the water and decreases the photosynthetic action. In addition, some dyes are toxic and carcinogenic, and thus, their treatment cannot only depend on biodegradation [3]. Methylene blue (MB), a cationic dye, is used extensively in the textile industry for dyeing wool, cotton, and silk, and as a pollutant in wastewater can cause vomiting, diarrhoea, nausea, and a burning sensation in the eyes [4]. Photocatalytic degradation of dyes is one of the most effective methods for water treatment [5]. Photocatalysts have been applied to purify polluted water, tiles, and air via titanium oxide materials with unique self-cleaning glasses, photo-induced super hydrophilicity, and titanium implants. TiO_2_ in different types and forms is one of the most widely used photocatalysts in the water and wastewater field because of its chemical stability, powerful oxidising power, non-toxicity, low cost, and biocompatibility [6]. However, bare TiO_2_ has some limitations including the high rate of the electron-hole recombination of the photo-generated charge carriers [7], low adsorption capacity, and wide bandgap (approximately 3.2 eV); hence, it can only be used under UV light to produce electron-hole pairs [8]. Moreover, as only 5% of the solar radiation is UV light, TiO_2_ is not an efficient photocatalyst [9]. The low adsorption of organic poisonous contaminants and a small surface area decrease the TiO_2_ degradation efficiency in practical applications [10]; thus, research is ongoing to develop an appropriate method to separate the photo-induced charge of TiO_2_ and delay the charge recombination.

Waste valorisation is the use of biomass materials, including residues such as forestry, agricultural, and industrial waste, as precursors. Carbonaceous materials such as graphite, activated carbon, porous carbon, carbon nanotubes, and graphene oxide, due to their unique characteristics in environmental applications, are the most important ingredients for TiO_2_ [9]. Recently, Khalid et al. (2017) [11] and Awfa et al. (2018) [12] reviewed the use of TiO_2_ modification with carbonaceous materials as an innovative and alternative approach to improve photocatalytic treatment. Among carbonaceous materials, activated carbons (ACs) are low cost [13,14], with high porosity and surface area (500–3000 m^2^/g, in the whole range of micro- (<2 nm), meso- (2–50 nm), and macropores (>50 nm), high charge carrier mobility, as well as textural properties [14]. TiO_2_ supported on AC has been adopted in the removal of organic contaminants because of the formation of a crystalline framework to support TiO_2_ nanoparticles, thus providing a useful approach to overcome the TiO_2_-related drawbacks [15]. Carbon-based composites can accept photo-generated electrons from the photocatalytic TiO_2_ nanoparticles, promoting a rapid photo-induced charge separation and a slow charge recombination. In general, carbonaceous materials with TiO_2_ composites show higher adsorption capacity, photocatalytic activity, sensitisation ability, scavenging of electrons, and extended absorption of visible light relative to bare TiO_2_ [11]. Furthermore, studies on the use of photocatalytic AC–TiO_2_ for the photo-degradation of water impurities (such as dyes) are very limited.

Magnetically recoverable suspended photocatalysts form the bridge between super photo-catalytic efficiency and fast recovery and the reusability of the photocatalyst after degradation. Easy photocatalyst separation after the treatment of water via an external magnetic field and high catalytic activity under UV/visible light can be achieved using magnetic photocatalysts [16]. Nowadays, the synthesis of recoverable magnetic materials for environmental remediation applications has gained considerable attention because of their unique advantages [17,18]. Fe_3_O_4_ loaded on AC has been explored as a catalytic activity improvement [19] and highly effective heterogeneous Fenton catalyst for the degradation of organic pollutants because of the significant promotion of H_2_O_2_ decomposition [20].

In this work, we investigated the comparative dosage ratio of synthesised AC/Fe_3_O_4_/TiO_2_ to obtain novel photocatalysts for the degradation of pollutants. Moreover, the effects of synthesised AC, TiO_2_, magnetic AC, initial pH, and oxidation by H_2_O_2_ on the degradation of MB and reusability were investigated. To the best of our knowledge, the literature on the formation of a TiO_2_/AC/Fe_3_O_4_ catalyst via a simple method for the improvement of MB degradation is still lacking; therefore, this study aimed to optimise the catalytic activity in an MB solution to improve its degradation and reusability performance. Synthesised porous AC and magnetic particles of Fe_3_O_4_ were used to improve the TiO_2_ photocatalytic activity and reusability applications by enlarging the TiO_2_ absorption region, increasing its surface area, and enhancing the stability of the catalyst during the regeneration process. Such a mechanistic understanding is very important for the controlled growth of TiO_2_/AC/Fe_3_O_4_, which may be used in many applications.

## 2. Experimental

### 2.1. Preparation of AC and Fe_3_O_4_/AC

A coconut shell was used to synthesise AC in a simple step. The coconut shell was washed with distilled water to remove impurities, then oven-dried at 70 °C for 10 h. The dried coconut shell was ground and placed in a tube furnace at 800 °C for 2 h, with a heating rate of 10 °C/min under a purified nitrogen flow. The prepared AC was washed several times to remove the smell and dried in an oven at 70 °C for 1 day for further experiments.

Ferrous and ferric phosphate salts in an alkaline aqueous solution were used for the fabrication of Fe_3_O_4_/AC nanoparticles via in situ chemical co-precipitation. Briefly, 5 g of AC, 6.66 g of FeCl_3_·6H_2_O (0.06 M), and 3.66 g of FeSO_4_·7H_2_O (0.04 M) were dissolved in 200 mL of distilled water and stirred vigorously with a mechanical stirrer on the hot plate and heated at 85 °C ± 1 °C for 1 h. Then, a KOH solution (20 M) was added dropwise into the prepared mixture while stirring with a magnetic stirrer until the pH reached 10–11. The mixture was stirred for 1 h to precipitate the hydrated iron oxide and cooled to room temperature. A strong magnet was used to separate the black precipitate, which was repeatedly rinsed with deionised water seven times and finally dried at 75 °C overnight in an oven.

### 2.2. Preparation of Fe_3_O_4_/AC/TiO_2_

The desired amount of the prepared and dried magnetic AC (1:1, 1:2, 1:4, and 1:8 Fe_3_O_4_/AC:TiO_2_ molar ratio) was obtained as follows: first, TiO_2_ nanoparticles were dissolved in ethanol and homogenised by ultrasonication for 10 min. The obtained Fe_3_O_4_/AC was added to the solution and mixed on the hot plate at 110 °C for 1 h (during the heating, most of the solution evaporated) before the mixture was calcined at 400 °C in the furnace (without washing). The calcined catalysts were washed with distilled water seven times and oven-dried at 100 °C for 24 h.

### 2.3. Catalyst Characterisation

X-ray diffraction (XRD) was conducted using a Bruker D8 Advance X-Ray diffractometer operating at (40 kV, 35 mA) under Cu–Kα radiation (λ = 0.154 nm). Scanning electron microscopy (SEM) was performed with an FE-SEM, JEO JSM 7600-F (JOEL Ltd., Tokyo, Japan) instrument equipped with an EDX. Thermogravimetric analysis (TGA) was conducted on a Mettler Toledo TGA/SDTA 851e (Mettler Toledo Corporation, Zurich, Switzerland) and heated from room temperature to 1000 °C at a heating rate of 10 °C/min in air. The Raman spectra were collected by a Renishaw model 1000 Raman microscope (Gloucestershire, UK) using an excitation wavelength of 514 nm in an ambient environment.

### 2.4. Photo-Catalytic Activity for MB Degradation

MB as the case organic pollution was selected for the photo-degradation experiment to investigate the UV-assisted degradation of the MB solution by synthesised AC, Fe_3_O_4_/AC /TiO_2_, Fe_3_O_4_/AC, and TiO_2_ at room temperature using a 1000-W UV lamp which emitted light with a wavelength of 664 nm. All the reactions were performed in magnetically stirred glass vessels located at a distance of 5 cm from the UV lamp and open at the top. Next, 0.1 g of the catalyst was added to 100 mL of the MB dye (100 mg/L, pH 11), then the mixture was sonicated in a water bath for 30 min to ensure MB adsorption equilibrium on the catalyst surface (120 rpm in the dark place). The solution was irradiated to degrade MB in the dark to prevent the impact of the outer light. The MB concentration of all the solutions was measured using a UV–VIS spectrophotometer (Shimadzu UV-2700 UV-Vis, Shimadzu, Kyoto, Tokyo) by measuring the absorbance of the solution (λ = 664 nm). Moreover, the effect of the initial pH in the range of 10–13 on the photo-catalyst activity of Fe_3_O_4_/AC/TiO_2_ 1:2 was investigated. The effect of H_2_O_2_ on the degradation of the MB dye over time was investigated by adding 0.1 g of 40 mM H_2_O_2_ to the dye solution as an oxidising agent. All the experiments were performed in triplicate. The removal efficiency (%) of MB was calculated using the following equation:

(1) $$removal\;\%=\frac{C_{0}-C_{t}}{C_{0}}\times 100$$

where *C*_0_ is the initial concentration of MB and *C_t_* is the MB concentration at different irradiation times. Furthermore, the reusability potential and the stability of the catalyst were investigated for seven cycles.

## 3. Results and Discussion

### 3.1. XRD Measurements of Fe_3_O_4_/AC and Fe_3_O_4_/AC/TiO_2_

**Figure 1:**
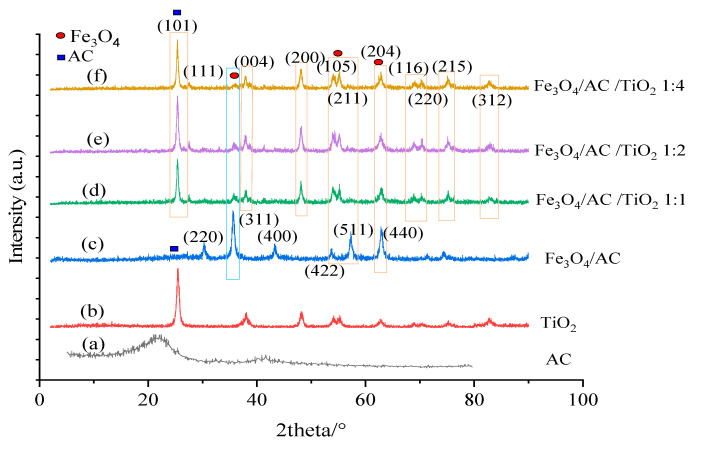
XRD patterns of AC (**a**), TiO_2_ (**b**), Fe_3_O_4_/ AC (**c**), Fe_3_O_4_/AC/TiO_2_ prepared at different ratios of 1:1 (**d**) to 1:2 (**e**) and 1:4 (**f**).

The structural properties of the prepared composites were characterised by X-ray diffraction, with the typical XRD patterns for the synthesised AC, TiO_2_ nanoparticles, Fe_3_O_4_/AC, and Fe_3_O_4_/AC/TiO_2_ in different ratios shown in Figure 1. In Figure 1a, the peaks at 24.8° and 42.5° denote the carbonaceous structures in the AC [21]; Figure 1b shows the XRD spectrum of TiO_2_ nanoparticles, and Figure 1c shows the XRD spectrum of Fe_3_O_4_/AC with six relatively intense peaks at 2θ values of 30.28° (220), 35.44° (311), 43.48° (400), 53.64° (422), 57.3° (511), and 62.6° (440) belonging to the cubic phase diffraction of the orthorhombic magnetite (JCPDS no. 19e0629) [22]. Figure 1c also shows a weak peak at 2θ = 25.1° corresponding to AC. The XRD peaks of Fe_3_O_4_/AC/TiO_2_ (Figure 1d–f) in different ratios at 2θ were 25.4° (101), 27.5° (111), 37.88° (004), 48.14° (200), 53.8° (105), 55.12° (211), 62.8° (204), 68.9° (116), 70.3° (220), 75.28° (215), and 82.7° (312), which were in good agreement with the JCPDS data of anatase TiO_2_. In addition, peaks appearing at 2θ = 35.44°, 53.8°, and 62.8° reflected the magnetic particles.

There was a strong peak at 25.4° that belonged to the (101) reflection of anatase. The sizes of the particles could be calculated with the anatase diffraction peaks (101) according to Scherrer’s equation: D = Kλ/(β cosθ) (K = 0.89 and λ = 0.154056 nm), where β is the full width at half maximum (FWHM), and θ denotes Bragg’s angle. The crystalline size of Fe_3_O_4_/AC and Fe_3_O_4_/AC/TiO_2_ (1:1), (1:2), and (1:4) was approximately 14, 18.7, 18.3, and 18.9 nm, respectively, indicating that increasing the ratio of TiO_2_ from (1:1) to (1:2) decreased the crystallite size, which then increased when the TiO_2_ ratio was increased from (1:2) to (1:4). In addition, increasing the ratio from (1:1) to (1:4) decreased the presence of the (311) peak, which was attributed to the elimination of the magnetic properties of the samples upon the increase in the TiO_2_ ratio. However, increasing the TiO_2_ ratio had no significant effects on the particle size. Moreover, TiO_2_-coated carbon-based materials (d–f) showed the same diffraction peaks as TiO_2_, with a small difference in the peak width corresponding to the increase in the crystallite size.

### 3.2. Surface Morphology Analysis of Fe_3_O_4_/AC/TiO_2_ and Fe_3_O_4_/AC

**Figure 2:**
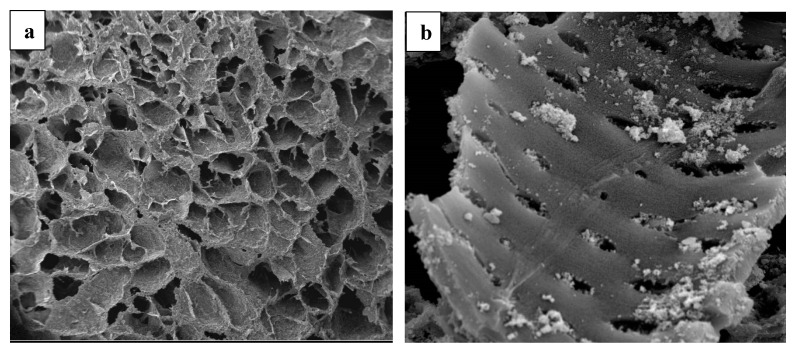
SEM images of (**a**) synthesised AC and (**b**) Fe_3_O_4_/AC.

The morphology of the synthesised coconut shell AC was observed by SEM (Figure 2a), showing the AC porous structure and heterogeneous surface. The pores observed (size: several micrometres) acted as channels for the adsorbents entering the adsorbate entrance. Figure 2b shows the presence of white aggregates of the metal oxide in the pores of the tile-like AC structures.

**Figure 3:**
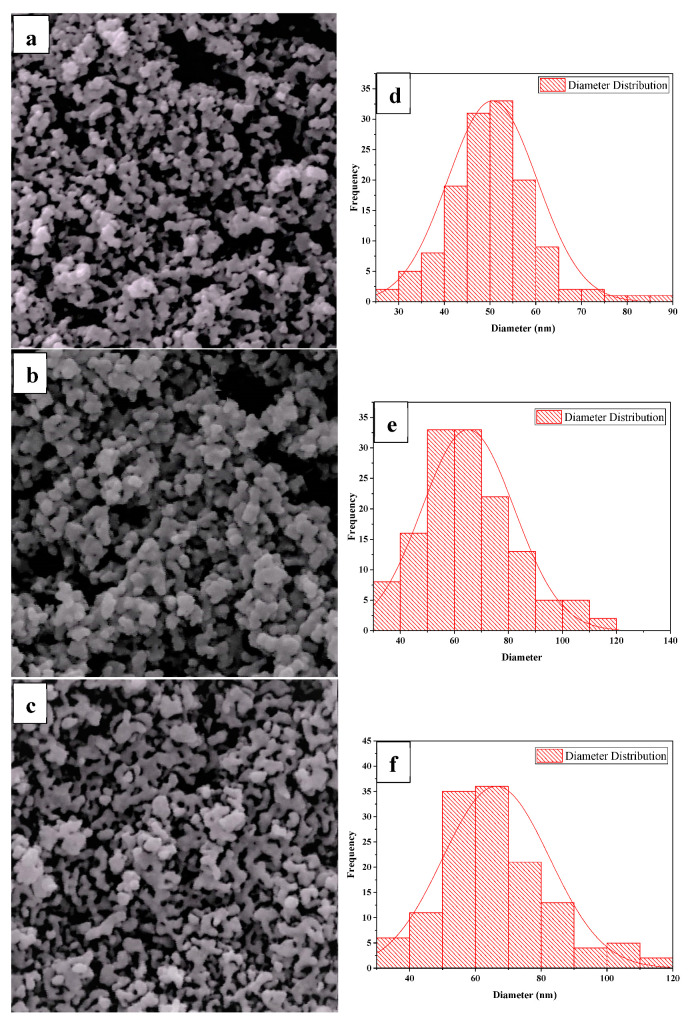
SEM images of Fe_3_O_4_/AC/TiO_2_: (**a**) 1:1, (**b**) 1:2, (**c**) 1:4, and (**d**–**f**) corresponding particle size histograms.

The SEM results of the TiO_2_-coated Fe_3_O_4_/AC samples are presented in Figure 3a–c. The TiO_2_ coatings were expected to modify the morphology of carbon, resulting in almost spherical particles. The ImageJ digital processing software was used to analyse the particle size and particle size distributions are shown in Figure 3d–f. The SEM analysis showed rough surfaces lightly produced on AC due to the loading of the TiO_2_ nanoparticles on the AC surface after synthesis. The TiO_2_ nanoparticles completely covered the AC surface without a reunion of its structure, even if it was not homogeneous. Figure 3 shows that TiO_2_ caused an increase in the average particle size of carbon, the average particle size was 50.22, 62.342, and 45.31 nm for sample 1:1, sample 1:2, and sample 1:4, respectively. As shown in Figure 3, the particle size increased with an increase in the TiO_2_ ratio from 1:1 to 1:2, then decreased with an increase in the ratio from 1:2 to 1:4, which was in good agreement with the obtained particle size from XRD. In Figure 3a,b, the TiO_2_ particles covered the Fe_3_O_4_/AC surface but were aggregated in Figure 3c.

**Figure 4:**
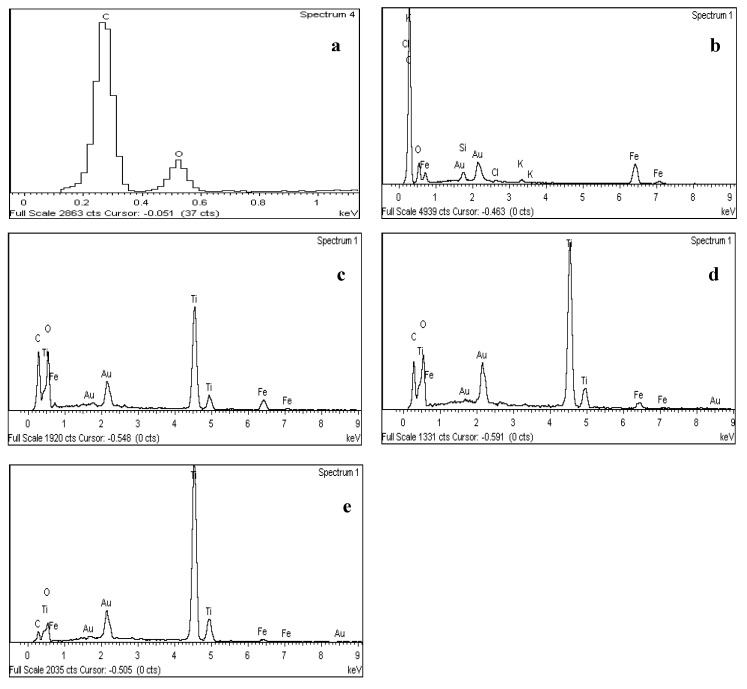
EDX spectra of (**a**) synthesised AC and (**b**) Fe_3_O_4_/AC and Fe_3_O_4_/AC/TiO_2_ in different ratios of (**c**) 1:1, (**d**) 1:2, and (**e**) 1:4.

The EDX spectra of the synthesised AC, Fe_3_O_4_/AC, and Fe_3_O_4_/AC/TiO_2_ in different ratios are shown in Figure 4a–e. Figure 4b indicates the presence of C, Fe, and O; Figure 4b shows the presence of potassium in the AC, which could be attributed to the addition of the KOH solution increasing the pH to 10–11. In Figure 4c–e, the peak ratio of Ti to AC and Fe confirmed the ratio of the used TiO_2_ and Fe_3_O_4_/AC. The presence of Au was attributed to the gold coating for SEM characterisation.

**Figure 5:**
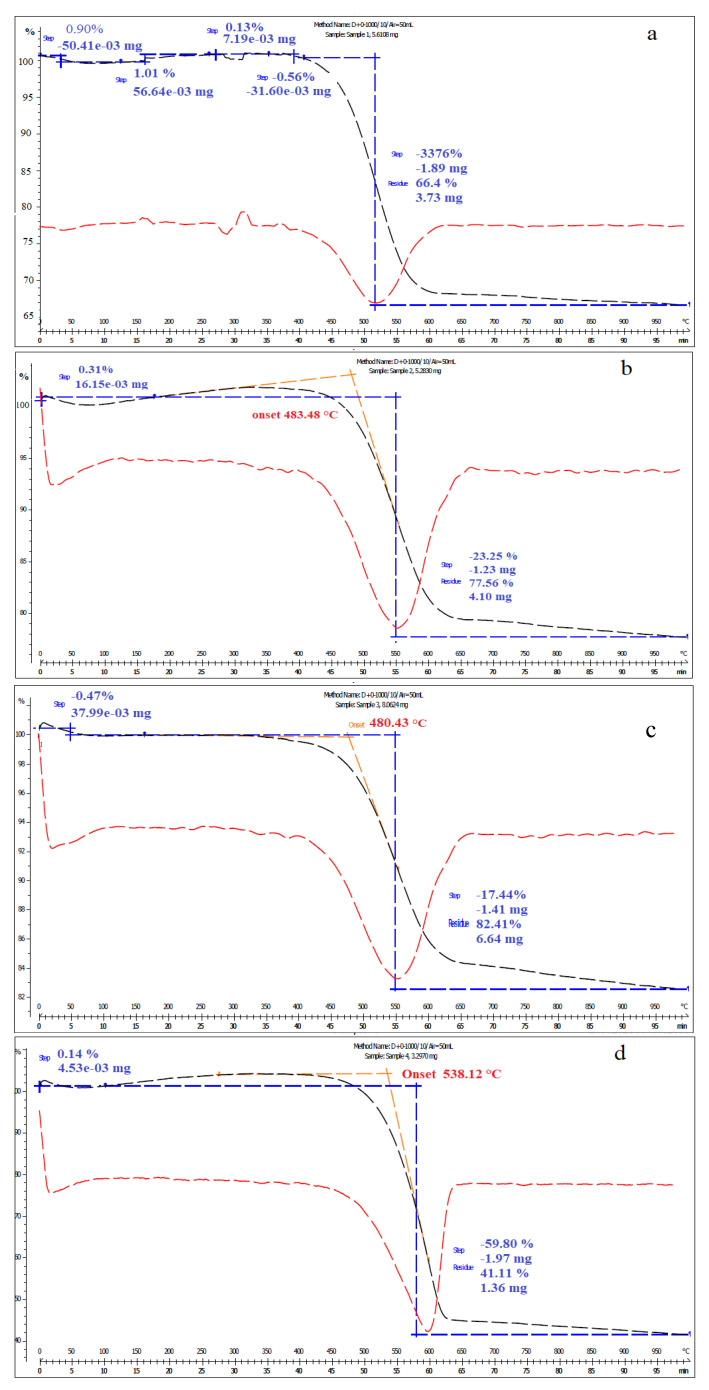
TGA curves of (**a**) Fe_3_O_4_/AC and Fe_3_O_4_/AC/TiO_2_ in ratios of (**b**) 1:1, (**c**) 1:2, and (**d**) 1:4 in an air atmosphere.

The TGA profiles in air for the samples of Fe_3_O_4_/AC and Fe_3_O_4_/AC/TiO_2_ in the ratios of 1:1, 1:2, and 1:4 are shown in Figure 5. Figure 5a shows that there was a one-step weight loss process. Fe_3_O_4_/AC was stable in air up to 538 °C, with the main weight loss occurring from 538 °C to 650 °C, and 59.8% weight loss until 1000 °C. The TGA measurements shown in Figure 5a indicate that the organic phases decomposed at temperatures below 650 °C. The TGA curves in Figure 5b–d show 33.76%, 23.25%, and 17.44% weight loss of Fe_3_O_4_/AC/TiO_2_ (1:1), (1:2), and (1:4), respectively. Increasing the ratio of TiO_2_ caused a decrease in the weight loss percentage, indicating that a significant loss of carbon from AC (in Fe_3_O_4_/AC/TiO_2_) did not occur and that carbon might be inserted within the TiO_2_ structure, thereby confirming that TiO_2_-loaded AC materials had better adsorption capacity and photocatalyst ability at higher temperatures. Meanwhile, no exothermic peaks were observed in either of the TGA curves (Figure 5b–d) at around 450 °C, indicating that there was no brookite transformation to the anatase phase in the Fe_3_O_4_/AC/TiO_2_ samples [23,24], so the brookite phase became more stable during the calcination of the samples at 400 °C. Therefore, the Fe_3_O_4_/AC/TiO_2_ composite was thermally stable, hence suitable for the practical applications.

**Figure 6:**
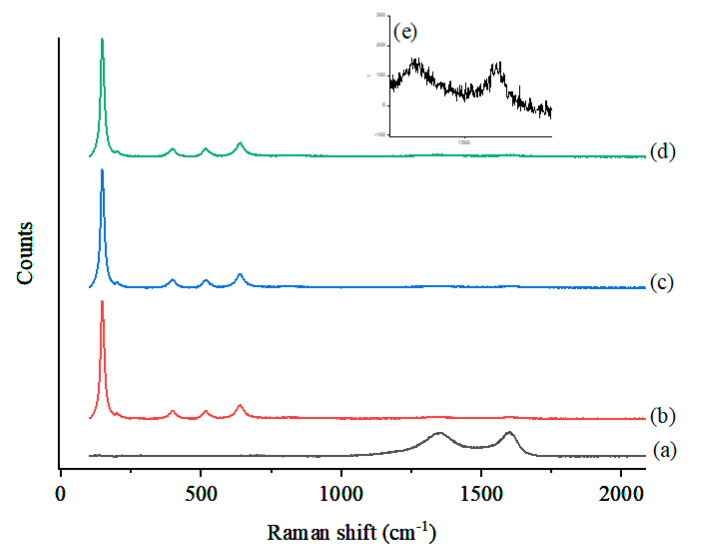
Raman spectra of (**a**) Fe_3_O_4_/AC and Fe_3_O_4_/AC /TiO_2_ in ratios of (**b**) 1:1, (**c**) 1:2, and (**d**) 1:4.

The Raman spectra of the obtained samples were collected in the range of 0–2000 cm^−1^ and are presented in Figure 6. Figure 6a shows two diffraction peaks around 1325 cm^−1^ (G band) and 1582 cm^−1^ (D band). The D band of the carbon material structure was associated with defects and became active when the crystallinity decreased, and the G band corresponded to the stretching vibrations with the basal graphene layers [25]. The characteristic diffraction peak around 670 cm^−1^ corresponded to Fe_3_O_4_ and demonstrated the magnetic property of the obtained sample. The combination of Raman and XRD findings confirmed that the Fe_3_O_4_ composite was formed during the synthesis. Figure 6b–d shows a comparison of the Raman spectra of the Fe_3_O_4_/AC/TiO_2_ composites in the ratio of 1:1, 1:2, and 1:4, respectively. A well-resolved TiO_2_ Raman peak was observed at ~149 cm^−1^ and was attributed to the main E_g_ anatase vibration mode for all three samples. The three diffraction peaks observed at around 396, 515, and 639 cm^−1^ indicated the major species of anatase crystallites [24]. There were two broad and weak peaks (in all the three Fe_3_O_4_/AC /TiO_2_ samples) at ~1300 cm^−1^ and 1600 cm^−1^, which were assigned to the ill-organised graphite and E_2g_ mode in graphite, respectively (Figure 6e) [26].

### 3.3. Photo-Catalytic Activity

**Figure 7:**
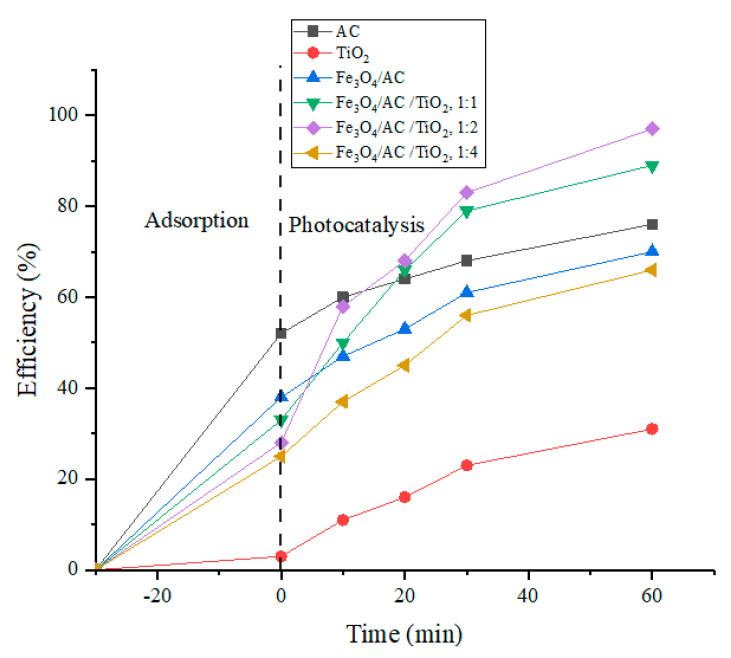
Methylene blue (MB) removal efficiencies over catalysts (AC, TiO_2_, Fe_3_O_4_/AC, Fe_3_O_4_/AC/TiO_2_ (1:1, 1:2, 1:4)) under UV light.

The synthesised catalyst activities were evaluated based on the photo-degradation of the MB aqueous solution (100 mg/L, pH 11) under UV irradiation at 664 nm. A blank experiment with the MB dye solution and with no photocatalyst was performed for comparison. After 30 min of irradiation in the absence of the photocatalyst, no evident MB degradation was observed. The pure photocatalytic removal efficiencies of the commercial TiO_2_ nanoparticles (in 30 min and 60 min) were 23% and 31%, respectively. The adsorption efficiencies of the synthesised coconut shell AC were 68% and 76.2% (in 30 min and 60 min) and the Fe_3_O_4_/AC/TiO_2_ photocatalyst samples presented a high removal efficiency (ranging from ~66% (1:4) to 98% (1:2)). TiO_2_ loaded on AC presented a very high photocatalytic degradation efficiency compared with pure TiO_2_, synthesised AC, and Fe_3_O_4_/AC (Figure 7). The concentration of the MB aqueous solution decreased significantly, in the two-step physical-chemical phenomenon of (1) adsorption by AC and (2) photocatalytic decomposition by TiO_2_ and Fe_3_O_4_. The magnetic AC played the role of an adsorbent at the ratios of 1:1, 1:2, and 1:4 (Fe_3_O_4_/AC and TiO_2_), with most AC channels not dominated by the TiO_2_ [10]. Figure 7 shows a very low degradation percentage of the photocatalyst samples of Fe_3_O_4_/AC and TiO_2_ in the ratio of 1:4, which may be attributed to the aggregation of TiO_2_ on AC, as shown in Figure 3c. The adsorption mainly occurred on the surface of the catalysts, and AC played the main role of an adsorbent, with the degradation subsequently occurring on TiO_2_ [27]. The porous structure of AC with an appropriate content resulted in the dye molecules gathering around the TiO_2_ nanoparticles at a low concentration of the MB solution. Therefore, the porous structure of AC was very important in facilitating the diffusion of the MB reactants and products on the TiO_2_ active sites during the photocatalytic reaction, which improved the photocatalytic degradation process [7].

**Figure 8:**
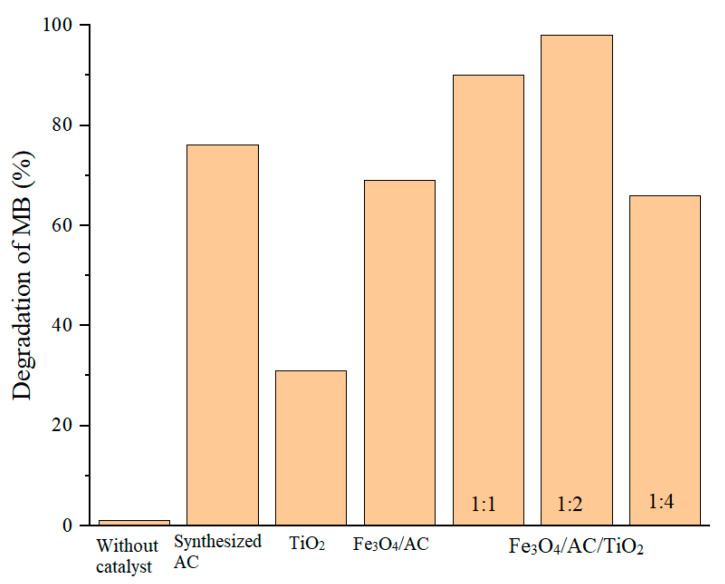
Photocatalytic degradation of MB for pure TiO_2_, synthesised AC, Fe_3_O_4_/AC, and Fe_3_O_4_/AC/TiO_2_ (1:1, 1:2, 1:4).

Figure 8 presents the result of the degradation of MB for pure TiO_2_, synthesised AC, Fe_3_O_4_/AC, and Fe_3_O_4_/AC /TiO_2_ (1:1, 1:2, 1:4). The degradation of MB without catalyst was used as a reference. The results show that: without the catalyst, no MB is degraded under UV-light, implying MB is relative stability under irradiation. In the presence of catalysts, the MB-degradation efficiency is greatly improved [28,29].

The degradation performance of the Fe_3_O_4_/AC /TiO_2_ catalysts with different initial pH values (10–13) was investigated. Increasing the pH from 10 to 12 increased the degradation performance from ~91.4% to 98.6%, the degradation performance decreased at pH 13 to ~84% after 120 min. The maximum value of the catalytic activity was observed at pH 12 (~98.3%). These results indicated that the pH significantly affected the degradation efficiency of the catalyst, which was heavily dependent on the transformation of the surface properties and activities of the catalyst and the pollutant [30,31].

**Figure 9:**
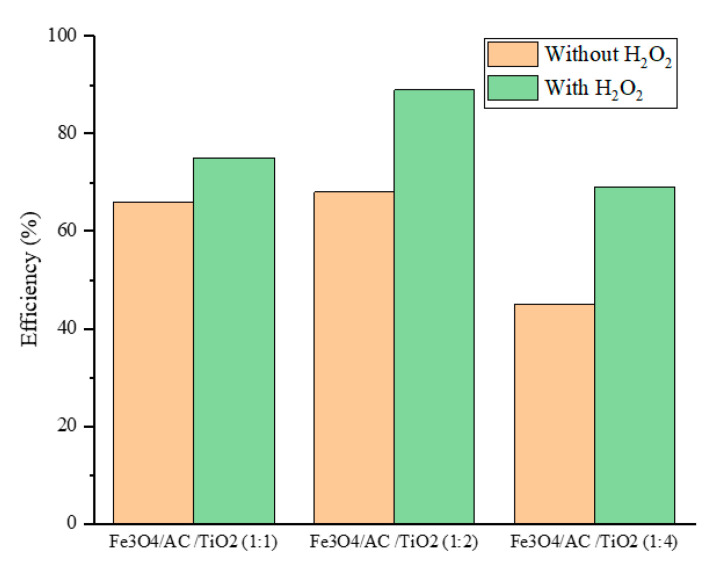
Effect of H_2_O_2_ on the removal efficiency of MB under UV light at pH 12.

In addition, the presence of H_2_O_2_ during the MB photocatalytic degradation was crucial. In this study, the MB photocatalytic degradation was evaluated using Fe_3_O_4_/AC/TiO_2_ (best photocatalyst) with H_2_O_2_ and with free H_2_O_2_. Figure 9 shows that the presence of H_2_O_2_ accelerated the photocatalytic performance of the nanocomposites, which could be due to the production of the active hydroxyl radicals. Hydrogen peroxide, as a powerful oxidiser, promoted the photocatalytic performance of the nanocomposites [32]. As reported by Poulopoulos et al. [33], a combination of H_2_O_2_ with TiO_2_ is very effective in improving the photocatalytic performance.

## 4. Reusability of Catalyst

**Figure 10:**
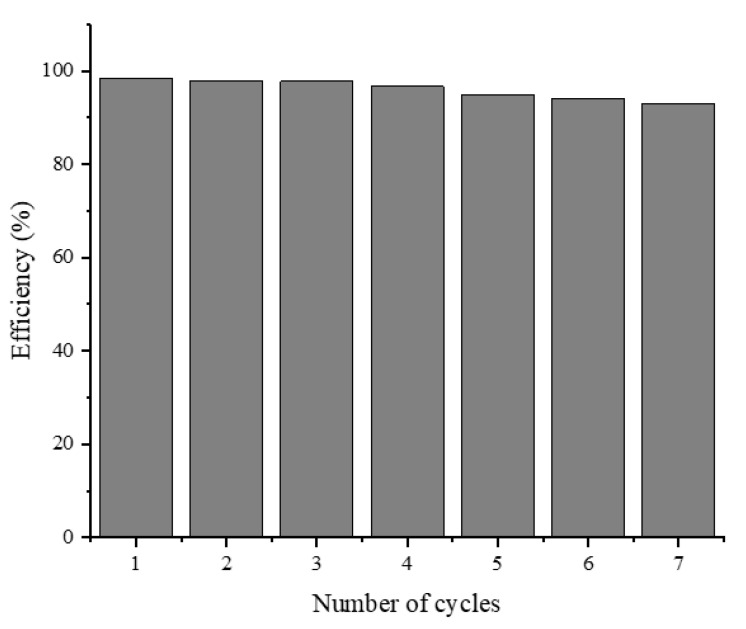
Recycling potential of Fe_3_O_4_/AC/TiO_2_ (1:2) for catalytic degradation during the 60-min reaction.

**Figure 11:**
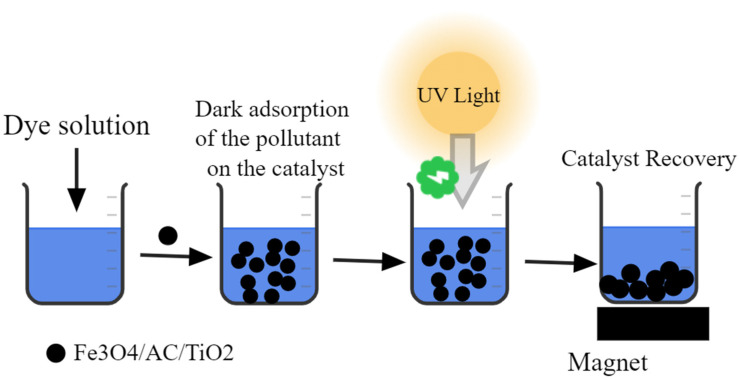
Recovery methodology after the photo-catalytic treatment of the pollutant solution.

Recovery and reusability are essential parameters for the selection of a cost-effective and feasible catalyst for pilot-scale remediation systems. The reusability performance of our best nanocomposite was investigated for seven cycles of MB photo-degradation using 100 mL of the MB dye (100 mg/L). Figure 10 shows the recyclability and stability of the Fe_3_O_4_/AC/TiO_2_ (1:2) catalyst after seven cycles during the 60-min reaction. The Fe_3_O_4_/AC/TiO_2_ catalyst could be recycled conveniently after the treatment via a strong magnet and reused; the results are depicted in Figure 10. The catalyst recovery and reusability processes are shown in Figure 11. After seven cycles, the photo-activity of Fe_3_O_4_/AC/TiO_2_ decreased from ~98% to ~93% (only 5%) after seven successive cycles with high efficiency. This slight decrease in the photocatalysis activity could be attributed to the following: (1) material losses might occur during the recovery step (washing and drying), which would lead to a lower dose in the subsequent cycle, thereby decreasing the surface catalytic activity and degrading the performance [34]. (2) The properties of magnetic nanoparticles, such as aggregation (this effect can reduce the effective surface area and decrease the number of active sites) and fouling, might change during the seven cycles [35]. (3) The adsorptive catalytic surface activity of the catalyst gradually decreased because of the obstruction of the pores and the active sites by catechol and its intermediates after each cycle [30,36]. In general, several cycles can be conducted using the same material with almost the same pollutant degradation efficacy [37,38].

## 5. Conclusions

Coconut shell AC and magnetic Fe_3_O_4_/AC/TiO_2_ have been synthesized in this work. The samples were characterized via different techniques. All the synthesised samples showed higher degradation efficiency under UV light than commercial TiO_2_, in particular, Fe_3_O_4_/AC/TiO_2_ (1:2) presented the highest degradation rate of 98% in 60 min. The simple synthesised coconut shell AC showed high adsorption efficiency (76.2%) and could be used with the TiO_2_ photo-catalyst to enhance the TiO_2_ photocatalytic activity (22% higher). The prepared magnetic photocatalysts presented good magnetic separation efficiency, which made the recovery and reusability affordable and simple. In conclusion, this paper presents an environmentally friendly and economical alternative for the photo-catalyst degradation of MB dye using the widely available agricultural waste for the production of TiO_2_-loaded carbonaceous materials.
